# Longitudinal Assessment of Hematologic and Immunonutritional Biomarkers from Treatment Initiation to Progression in Metastatic Colorectal Cancer

**DOI:** 10.3390/biomedicines14040799

**Published:** 2026-04-01

**Authors:** Ljiljana Mayer, Ljubica Vazdar, Ana Tečić Vuger, Laura Mayer, Iva Andrašek, Sanja Langer, Ines Sever, Zvjezdana Špacir Prskalo, Milica Vrbančić, Mihaela Gaće, Robert Šeparović

**Affiliations:** 1 Department of Medical Biochemistry in Oncology, University Hospital for Tumors, Sestre Milosrdnice University Hospital Center, 10000 Zagreb, Croatia; ljiljana.mayer@kbcsm.hr (L.M.); sanja.langer@kbcsm.hr (S.L.); ines.sever@kbcsm.hr (I.S.); zvjezdana.spacir@kbcsm.hr (Z.Š.P.); milica.sostaric@kbcsm.hr (M.V.); mihaela.gace@kbcsm.hr (M.G.); 2 School of Medicine, Catholic University of Croatia, 10000 Zagreb, Croatia; 3 Division of Medical Oncology, University Hospital for Tumors, Sestre Milosrdnice University Hospital Center, 10000 Zagreb, Croatia; ljubica.vazdar@kbcsm.hr (L.V.); ana.tecic@kbcsm.hr (A.T.V.); 4 School of Medicine, University of Zagreb, 10000 Zagreb, Croatia; 5 Zabok Branch, Institute of Emergency of the Krapina-Zagorje County, 49000 Krapina, Croatia; laura.mayer.zg@gmail.com; 6 Department of Radiotherapy, University Hospital for Tumors, Sestre Milosrdnice University Hospital Center, 10000 Zagreb, Croatia; iva.andrasek@kbcsm.hr; 7 Faculty of Medicine, Josip Juraj Strossmayer University of Osijek, 31000 Osijek, Croatia; 8 Faculty of Medicine in Pula, Juraj Dobrila University in Pula, 52100 Pula, Croatia

**Keywords:** metastatic colorectal cancer, systemic immune-inflammation index, neutrophil-to-lymphocyte ratio, platelet-to-lymphocyte ratio, nutritional status, sarcopenia, longitudinal biomarkers

## Abstract

**Background**: Systemic inflammatory indices such as the neutrophil-to-lymphocyte ratio (NLR), platelet-to-lymphocyte ratio (PLR), and systemic immune-inflammation index (SII) have demonstrated prognostic relevance in metastatic colorectal cancer (mCRC). However, most available evidence relies on single baseline measurements, while the longitudinal dynamics of these biomarkers in relation to immunonutritional status remain insufficiently explored. **Methods**: This retrospective longitudinal study included 86 patients with previously untreated mCRC receiving first-line chemo-biological therapy. NLR, PLR, and SII were assessed at three predefined time points: before treatment initiation, after completion of induction therapy, and at radiologically confirmed disease progression. Nutritional and sarcopenia risk were evaluated using the NRS-2002 and SARC-F tools. Longitudinal differences were analyzed using the Friedman test with post hoc comparisons. **Results**: In nutritionally preserved patients, significant longitudinal changes were observed for NLR (χ^2^(2) = 16.72, *p* < 0.001), PLR (χ^2^(2) = 6.36, *p* = 0.003), and SII (χ^2^(2) = 24.57, *p* < 0.001), characterized by a marked decline following induction therapy and re-elevation at disease progression. In high nutritional risk patients, significant dynamics were observed only for SII (χ^2^(2) = 5.60, *p* = 0.007). Similarly, in the low SARC-F subgroup, all three indices demonstrated significant modulation over time, whereas no statistical analysis was feasible in the high SARC-F subgroup due to limited sample size. Among the evaluated parameters, SII showed the most consistent and pronounced longitudinal variation. **Conclusions**: The clinical value of inflammatory hematologic indices in mCRC appears to derive primarily from their longitudinal dynamics rather than single absolute measurements. SII, in particular, may serve as a marker of therapy-induced modulation of systemic inflammation, especially in patients with preserved immunonutritional reserve. Integration of dynamic inflammatory indices into routine clinical monitoring could enhance early identification of biological progression and improve risk stratification in mCRC.

## 1. Introduction

Colorectal cancer remains one of the most prevalent malignancies worldwide, with over 1.9 million new diagnoses each year, reflecting its substantial contribution to the global cancer burden [1]. According to recent data, Croatia ranks among the European countries with a higher colorectal cancer burden, with an incidence of approximately 75 new cases per 100,000 inhabitants and a mortality rate of about 32 deaths per 100,000 annually—both exceeding the respective European Union averages of roughly 65 and 25 per 100,000 [2]. Although well-structured screening programs have demonstrably reduced mortality where they are consistently implemented, outcomes for patients with metastatic disease remain poor, with five-year survival typically ranging from 10% to 15% [3]. The treatment of metastatic colorectal cancer (mCRC) is based on a multimodal approach that integrates cytotoxic chemotherapy, targeted agents and immunotherapy, with therapeutic choices largely determined by the tumor’s molecular profile, the primary tumor sidedness, the extent of metastatic spread and the patient’s overall clinical condition [4,5].

In metastatic colorectal cancer, treatment response is traditionally assessed using radiological criteria such as RECIST (Response Evaluation Criteria in Solid Tumors), together with clinical evaluation and serum tumor markers like CEA [6,7]. However, these measures often lag behind the true biological behavior of the disease and may fail to capture early therapeutic resistance or progression. Consequently, readily available hematologic, inflammatory and nutritional biomarkers derived from routine blood tests have gained increasing attention as dynamic indicators of treatment efficacy [8,9]. When assessed longitudinally from treatment initiation through disease progression, these markers may capture subtle shifts in tumor–host interactions before they become clinically or radiologically evident.

In this setting, systemic inflammation and immune dysfunction emerge as key drivers of disease progression and therapeutic resistance in mCRC, making simple hematologic inflammatory indices the subject of growing scientific interest [10,11,12]. Indices such as the neutrophil-to-lymphocyte ratio (NLR), platelet-to-lymphocyte ratio (PLR) and the systemic immune-inflammation index (SII) provide an integrated reflection of pro-tumor inflammatory activity and antitumor immune response. Longitudinal evaluation of these parameters enables differentiation between transiently modulated immunoinflammatory responses and persistently activated systemic inflammation, with direct clinical implications in mCRC.

Therefore, the aim of this study was to longitudinally assess changes in NLR, PLR and SII from treatment initiation to disease progression in patients with metastatic colorectal cancer, and to determine whether these changes can distinguish transient immunoinflammatory modulation from persistent systemic inflammation and provide clinically relevant information in relation to nutritional and functional status.

## 2. Materials and Methods

A retrospective longitudinal cohort study was conducted, based on the analysis of medical records and laboratory findings, including complete blood count and biochemical parameters, in patients with mCRC who had not previously received treatment for systemic disease. The study was carried out at the University Hospital for Tumors, Sestre milosrdnice University Hospital Center. It included 86 hospitalized patients older than 18 years, with good performance status (ECOG 0–1), a histologically confirmed diagnosis of mCRC, who initiated systemic chemo-biological treatment between April 2019 and February 2025. The study was performed in line with the principles of Declaration of Helsinki. Approval was granted by the Ethics Committee of the Sestre milosrdnice University Hospital Center, Zagreb, Croatia (Class No. 003-06/21-03/040; date: 9 December 2021).

The analysis was performed at three key time points. The first time point was immediately prior to induction treatment with a chemotherapy doublet plus bevacizumab: FOLFIRI (irinotecan 180 mg/m^2^, leucovorin 200 mg/m^2^, 5-fluorouracil 400 mg/m^2^ IV bolus followed by 5-fluorouracil 600 mg/m^2^ over 22 h plus bevacizumab 5 mg/kg; frequency 14 days) or FOLFOX plus bevacizumab (oxaliplatin 85 mg/m^2^, leucovorin 200 mg/m^2^, 5-fluorouracil 400 mg/m^2^ IV bolus followed by 5-fluorouracil 600 mg/m^2^ over 22 h plus bevacizumab 5 mg/kg; frequency 14 days). The second time point encompassed the period after completion of eight cycles of induction therapy, at the initiation of maintenance therapy with capecitabine (1250 mg/m^2^ twice daily, days 1–14) in combination with bevacizumab (7.5 mg/kg); frequency 21 days. The third time point corresponded to radiological (MSCT) disease relapse occurring during maintenance therapy with capecitabine and bevacizumab. This time point was defined as the moment of radiologically confirmed disease progression, and laboratory values were determined few days prior or after MSCT. Longitudinal within-subject design enabled control of interindividual variability and reduced the impact of between-subject heterogeneity, including differences between two chemotherapy protocols (oxaliplatin-based FOLFOX or irinotecan-based FOLFIRI), with the possibility of residual effects from uncontrolled factors.

At all three time points, parameters of nutritional status and sarcopenia, as well as laboratory findings, were analyzed. In the assessment of nutritional risk and sarcopenia, patients were stratified into two groups: high-risk and low-risk. The risk of malnutrition was assessed using the ESPEN-recommended screening tool for identifying malnutrition risk in hospitalized patients, the NRS-2002 (Nutritional Risk Screening 2002). Patients with a score < 3 were classified as low risk, whereas a score ≥ 3 defined patients as being at high risk for the development of malnutrition. All high-risk patients received continuous oral enteral nutritional support. Data for a certain number of patients were missing at the third time point due to patients’ disease progression and outcome, which did not allow for complete follow-up.

The risk of sarcopenia was assessed using the SARC-F questionnaire, which identifies clinical features suggestive of sarcopenia, including reduced muscle strength, difficulty walking, rising from a chair, climbing stairs, and the occurrence of falls. A SARC-F score ≥ 4 indicates an increased risk of sarcopenia.

Responses to the NRS-2002 and SARC-F questionnaires were obtained through patient interviews and completed by healthcare professionals.

NLR, PLR and SII were calculated based on hematological parameters measured routinely in the hospital laboratory on automated hematology analyzer Sysmex XN-1000 (Sysmex Corporation, Kobe, Japan) as part of complete blood count (CBC): NLR = absolute neutrophil count/absolute lymphocyte count; PLR = platelet count/lymphocyte count; SII = (platelet count × absolute neutrophil count)/absolute lymphocyte count.

Statistical analysis was done in MedCalc software v23.4.5 (Ostend, Belgium). Data distribution was tested by Shapiro–Wilk test and Friedman test was used accordingly for data comparison at different measurement time points for NLR, PLR, and SII. No other corrections for multiple testing were applied.

## 3. Results

Study included 86 metastatic CRC patients with median age of 66 (min–max: 40–85).

Summary statistics for NLR, PLR, and SII across different measurement time points and stratified by NRS and SARC-F scores are presented in Table 1, Table 2 and Table 3.

**Figure 1:**
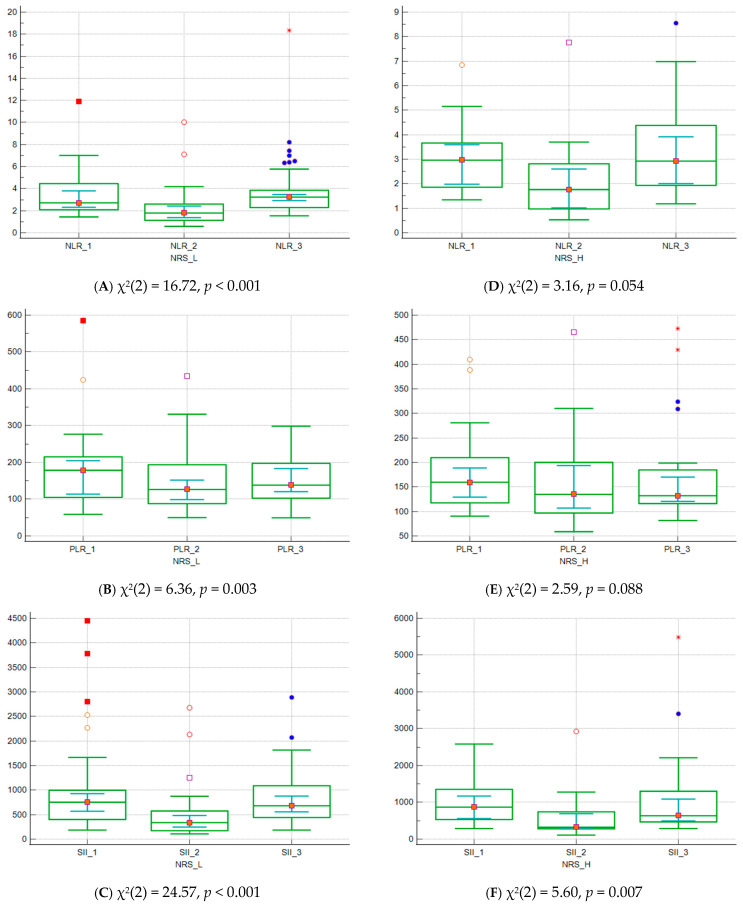
(**A**–**F**) Data comparison graphs for NLR, PLR, SII at different measurement time points for patients stratified into low- and high-NRS subgroups (Friedman test); central box represents the values from the lower to upper quartile (25 to 75 percentile). The middle line with red square in it represents the median. A line extends from the minimum to the maximum value, excluding outlier values which are displayed as separate points.

Data comparison graphs for NLR, PLR, SII at different measurement time points for patients stratified into low- and high-NRS subgroups are presented in Figure 1A–F. The Friedman test revealed statistically significant difference for all three calculated parameters in NRS-L subgroup and for SII in NRS-H subgroup. Post hoc analysis revealed that NLR, and SII values were lower in NRS-L subgroup at the end of chemotherapy in comparison to baseline values before initiating treatment protocol and at the time of disease relapse. For PLR, baseline values were significantly higher than after the treatment and the time of relapse. For NRS-H subgroup, SII was significantly lower after the treatment in comparison to baseline values, but were not different than values at the time of relapse.

**Figure 2:**
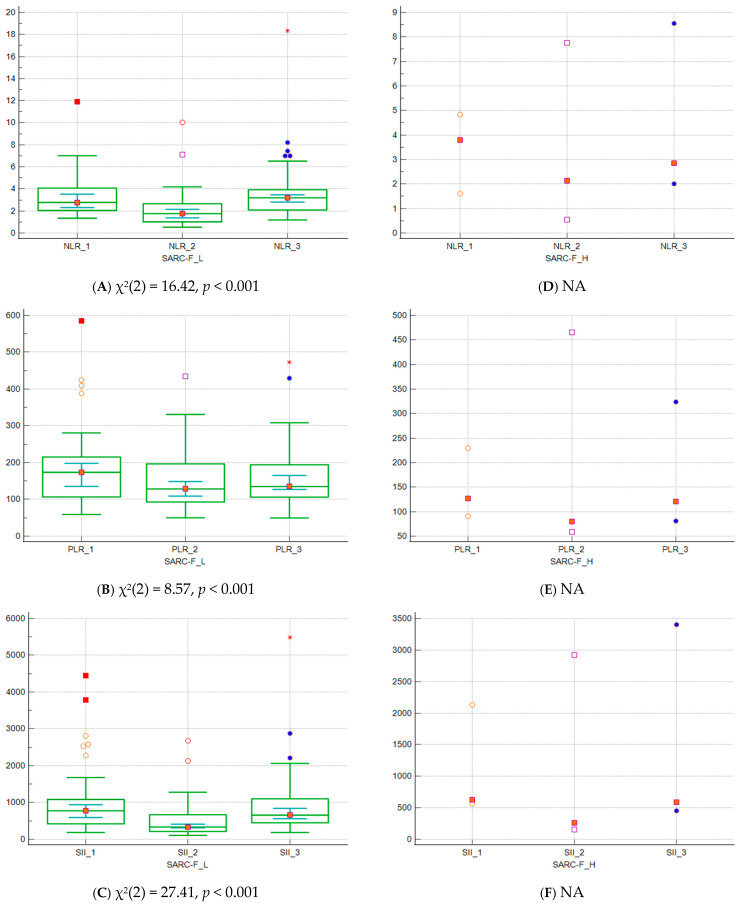
(**A**–**F**) Data comparison graphs for NLR, PLR, SII in different time points for patients in low and high SARC_F subgroup (Friedman test). NA–statistical analysis not applicable due to low number of participants; central box represents the values from the lower to upper quartile (25 to 75 percentile). The middle line with red square in it represents the median. A line extends from the minimum to the maximum value, excluding outlier values which are displayed as separate points.

Data comparison graphs for NLR, PLR, SII in different time points for patients in low and high SARC_F subgroup are presented in Figure 2A–F. The Friedman test determined statistically significant difference for all three calculated parameters in SARC_F-L subgroup. Post hoc analysis revealed that NLR values were lower after chemotherapy in comparison to baseline values and values at the time of disease relapse. For PLR, baseline values were significantly higher than after the treatment and the time of relapse. On the other hand, SII values were higher at baseline in comparison to both second and third time point. After the treatment they were the lowest, with a significant rise at the time of disease relapse. Number of patients in SARC_F-H subgroup was insufficient for statistical analysis and the figures show only isolated cases for each parameter.

## 4. Discussion

The present study contributes to the growing body of evidence on inflammatory biomarkers in metastatic colorectal cancer by providing a longitudinal evaluation of NLR, PLR and SII across clinically meaningful treatment phases. While most previous studies have focused primarily on baseline measurements, our findings highlight that the clinical relevance of these indices emerges predominantly from their dynamic changes during treatment rather than from single absolute values. Importantly, our study integrates inflammatory indices with clinically validated measures of nutritional and functional status (NRS-2002 and SARC-F), allowing a more comprehensive interpretation of tumor–host interactions.

Colorectal cancer continues to represent a significant public health burden in Croatia, with more than 3600 newly diagnosed cases annually. Despite the proven effectiveness of organized screening programs in reducing mortality, a substantial proportion of patients are still diagnosed at the metastatic stage of the disease, in which therapeutic options are limited and long-term outcomes remain unfavorable [13]. Unlike localized disease, where the primary therapeutic goal is cure, the management of mCRC is focused on prolonging survival, controlling tumor activity, and preserving patients’ functional status. Curative interventions may be considered only in strictly selected cases of oligometastatic disease, most commonly in patients with isolated hepatic or pulmonary metastases, where surgical resection or ablative approaches may be possible [14,15]. Assessment of treatment response in mCRC has traditionally relied on radiological criteria such as RECIST, in conjunction with clinical evaluation and monitoring of serum tumor markers [14]. However, these tools often fail to timely reflect the true biological dynamics of the disease and may delay the detection of early therapeutic resistance or biological progression. Consequently, there is a clear clinical need for additional, readily available biomarkers that could provide earlier insight into disease course and treatment efficacy.

In this context, hematological, inflammatory, and immunonutritional biomarkers derived from routine laboratory testing have increasingly been recognized as potential tools for dynamic monitoring of patients with mCRC. Their wide availability, rapid turnaround time, and suitability for repeated measurements make them particularly attractive for longitudinal follow-up, as they may reflect changes in systemic inflammation, immune competence and nutritional status that precede clinically or radiologically evident disease progression. While individual laboratory parameters such as neutrophil, lymphocyte, or platelet counts, C-reactive protein, or serum albumin provide valuable information on specific aspects of systemic inflammation, immune response, or nutritional status, their prognostic value is limited by their fragmented representation of the complex tumor–host interaction. In patients with mCRC, where the disease is characterized by the simultaneous activation of pro-inflammatory, immunosuppressive, and catabolic pathways, isolated parameters often fail to reliably capture the underlying biological dynamics [16,17,18].

Derived hematological inflammatory indices, such as NLR, PLR and SII integrate multiple biological components into a single numerical value, simultaneously encompassing inflammatory burden, immune competence, and hematopoietic activity. This integrative nature allows for a more comprehensive assessment of the patient’s systemic state and potentially more sensitive monitoring of changes associated with therapeutic response and biological disease progression. Owing to these characteristics, these indices are increasingly considered clinically relevant biomarkers in mCRC, particularly when evaluated longitudinally rather than as single baseline measurements.

Among complex inflammatory indices, NLR is one of the earliest and most extensively investigated biomarkers in colorectal cancer, particularly in the context of prognostic and clinical assessment in metastatic disease. Its prognostic relevance derives from its ability to integrate two biologically opposing processes into a single measure: pro-tumor inflammatory activation mediated by neutrophils and antitumor immune response predominantly reflected by lymphocytes. An elevated NLR therefore indicates a state in which tumor-promoting mechanisms overcome alongside compromised immune surveillance. Neutrophils contribute to angiogenesis, remodeling of the tumor microenvironment, and facilitation of invasion and metastatic dissemination, whereas lymphocytes, particularly T lymphocytes, represent key effectors of antitumor immunity. Thus, NLR does not merely represent a quantitative ratio of two cell populations, but rather a functional indicator of the balance between pro-tumor and antitumor processes [19,20].

Numerous retrospective and prospective studies, as well as meta-analyses, have consistently demonstrated that an elevated NLR is associated with poorer overall and progression-free survival in patients with colorectal cancer, including those with metastatic disease. In the mCRC setting, increased NLR is frequently interpreted as a reflection of a more aggressive tumor biology, heightened systemic inflammatory burden, and diminished host capacity to mount an effective therapeutic response. In many studies, its prognostic value remains independent of established clinicopathological factors. However, the majority of published investigations have relied on single baseline measurements, thereby neglecting the inherently dynamic nature of systemic inflammation and immune responses during the course of treatment [9,20,21,22].

Accumulating evidence indicates that longitudinal monitoring of NLR may provide clinically meaningful information beyond its initial absolute level. Longitudinal changes in NLR during therapy may reflect modulation of systemic inflammation as well as dynamic tumor–host interactions. Persistently elevated values suggest sustained pro-inflammatory activation associated with a more aggressive disease course and poorer prognosis. Importantly, NLR cannot be interpreted independently of patients’ nutritional and functional status, as malnutrition and sarcopenia are accompanied by chronic systemic inflammation and immune dysregulation [23,24,25].

In our cohort, statistically significant changes over time in NLR were observed exclusively in nutritionally and functionally preserved patient subgroups (NRS-L and SARC-F-L), characterized by a marked decline during treatment, and followed by re-elevation at the time of disease progression. In contrast, among patients with impaired nutritional and functional status (NRS-H and SARC-F-H), NLR remained persistently elevated without significant longitudinal oscillations, likely reflecting chronically activated systemic inflammation and limited biological reserve. These findings support the notion that the clinical relevance of NLR in mCRC derives predominantly from its dynamics over time, particularly when interpreted in conjunction with nutritional and functional parameters.

While NLR provides valuable insight into the balance between pro-inflammatory activity and antitumor immunity, PLR further emphasizes the role of platelets in tumor progression and represents a complementary indicator of systemic inflammation and immune dysfunction in mCRC. Platelets actively contribute to angiogenesis, shield circulating tumor cells from immune surveillance, and facilitate invasion and metastatic dissemination, whereas lymphopenia reflects compromised antitumor immunity [26,27,28].

Our results indicate that, similar to NLR, the clinical significance of PLR does not result from a single absolute measurement but rather from its dynamics during treatment. In patients who are nutritionally and functionally preserved (NRS-L and SARC-F-L), PLR demonstrated clear modulation, with a significant decrease during therapy and subsequent increase at progression, suggesting the presence of a biologically reversible inflammatory component responsive to therapeutic intervention.

In contrast, in patients with compromised nutritional and functional status (NRS-H and SARC-F-H), PLR remained persistently elevated without meaningful longitudinal variation, consistent with chronic systemic inflammation, immune dysfunction, and limited host reserve, which aligned with a more aggressive biological phenotype and poorer clinical outcomes.

These findings indicate that, although PLR provides meaningful biological insight, its interpretative value is limited when assessed as a standalone parameter. In contrast, its longitudinal evaluation, particularly when in context with the patient’s nutritional and functional status, yields more clinically relevant information regarding the dynamics of tumor–host interactions.

In contrast to individual ratios, SII integrates neutrophils, platelets, and lymphocytes. In our cohort, SII was the most sensitive indicator of dynamic changes during treatment. It reflected therapy-induced modulation of systemic inflammation in nutritionally and functionally preserved patients most consistently, with a pronounced decline during treatment and re-elevation at progression. This pattern suggests that SII more effectively captures the cumulative impact of pro-tumor and antitumor mechanisms and detects subtle biological shifts that may not be fully reflected by individual indices [29,30]. In our study design, the third time point was defined as the moment of radiologically confirmed disease progression, and laboratory values were determined at or immediately before this period. Due to this design, we were unable to systematically analyze the time interval between the increase in SII and radiological confirmation of progression. Therefore, although our results suggest that SII reflects the biological dynamics of the disease, we cannot conclude with certainty whether its increase precedes radiologically detectable progression by a clinically relevant time interval. Due to sample size and study design, we did not perform a separate analysis of patients who did not experience a decline in SII after induction therapy. However, the hypothesis that the absence of a decline in SII may represent an early marker of primary therapeutic resistance is interesting and biologically well-founded for future studies.

From a clinical perspective, these findings suggest that simple inflammatory indices obtained from routine blood tests may provide an early signal of biological progression before radiological changes become evident. In everyday oncology practice, longitudinal monitoring of SII in particular could serve as an inexpensive and easily accessible adjunct tool for dynamic patient assessment.

Several limitations of this study should be acknowledged. First, the retrospective single-center design may introduce selection bias and limit the generalizability of the findings. Second, the sample size of certain subgroups, particularly patients with high SARC-F scores, was relatively small, which restricted the possibility of performing statistical analyses in this group. Consequently, no firm conclusions can be drawn for this group and that the results are only preliminary. This fact points to an important gap in the literature and the need for future research specifically targeting this vulnerable population. Third, our study was not primarily designed or statistically powered to analyze survival outcomes (PFS or OS), but rather to longitudinally assess the biological dynamics of inflammatory indices at predefined time points during treatment. In addition, the relatively limited number of subjects, especially after stratification into subgroups (NRS, SARC-F), significantly limits the reliability of multivariate models that would include a larger number of clinicopathological variables (e.g., tumor location, RAS/BRAF status, extent of metastatic disease). Future studies with larger cohorts that include survival analysis are necessary to confirm the independent prognostic value of these biomarkers. Fourth, the inclusion of objective methods for body composition assessment (e.g., CT analysis) would further strengthen the findings. Finally, inflammatory indices may be influenced by conditions unrelated to cancer, such as infection or other inflammatory states. A review of the medical records was performed, and, wherever possible, time points associated with acute clinical conditions that could significantly influence inflammatory parameters (e.g., infections, thromboembolic events or other acute inflammatory conditions) were excluded from the analysis. Nevertheless, we are aware that due to the retrospective design, it is not possible to completely exclude the influence of subclinical or undocumented inflammatory events. Therefore, larger prospective studies are warranted to validate these findings and further clarify the clinical utility of longitudinal inflammatory biomarkers in metastatic colorectal cancer.

Future studies should explore whether integrating longitudinal inflammatory indices, particularly SII, with radiological and molecular markers could improve dynamic risk stratification and guide individualized treatment monitoring in metastatic colorectal cancer.

In accordance with the existing literature, our results confirm the clinical relevance of NLR, PLR, and particularly SII in metastatic colorectal cancer. However, they extend current knowledge by underscoring the importance of dynamics over time. Whereas prior studies and meta-analyses have predominantly focused on baseline values, our data indicate that treatment-associated changes in SII carry additional clinically meaningful information beyond the implications of single measurements. It is particularly noteworthy that significant longitudinal modulations of the SII were observed predominantly in patients with preserved nutritional and functional status, whereas in more vulnerable subgroups the values remained persistently elevated. This finding suggests that nutritional and functional status do not merely act as independent clinical factors, but also serve as key modulators of the capacity of the SII to reflect therapeutic response. Accordingly, the focus shifts from the question of whether inflammatory indices are clinically relevant to the question of when and in which patients they carry clinically meaningful information, supporting the contemporary concept of dynamic and context-dependent biomarker interpretation in mCRC.

## 5. Conclusions

In conclusion, this study demonstrates that the clinical potential of complex inflammatory hematologic indices in metastatic colorectal cancer does not arise from their single absolute measurement, but rather from their dynamics during treatment. Longitudinal monitoring of the NLR, PLR, and particularly the SII enables differentiation between patients with preserved immunonutritional and functional reserve, among whom systemic inflammation appears partially reversible and responsive to therapeutic intervention, and those with persistently activated systemic inflammation and a more unfavorable biological disease phenotype. Among the analyzed parameters, SII showed the most pronounced dynamics across time points, suggesting its potential clinical relevance, although confirmation in larger validation studies is warranted. Integration of these simple and readily available biomarkers into routine clinical monitoring of patients with mCRC may facilitate earlier identification of therapeutic resistance, more precise risk stratification, and greater individualization of treatment, especially when interpreted in the context of the patient’s nutritional and functional status.

## Figures and Tables

**Table 1:** Summary statistics of NLR values across different measurement time points and stratified by NRS and SARC-F scores.

NLR		1.	2.	3.
NRS-L (<3)	N	60	60	42
	Median	3.17	1.76	3.23
	(IQR)	(2.15–4.29)	(1.03–2.81)	(2.29–3.86)
NRS-H (≥3)	N	26	26	20
	Median	2.78	1.82	2.92
	(IQR)	(1.62–3.64)	(0.97–2.70)	(1.94–4.38)
SARC_F-L (<4)	N	82	82	59
	Median	3.02	1.73	3.20
	(IQR)	(2.08–4.12)	(0.97–2.80)	(2.09–3.93)
SARC_F-H (≥4)	N	4	4	3
	Median	3.24	2.21	2.86
	(IQR)	(2.16–4.31)	(1.34–5.03)	(2.22–7.12)

**Table 2:** Summary statistics of PLR values across different measurement time points and stratified by NRS and SARC-F scores.

PLR		1.	2.	3.
NRS-L (<3)	N	60	60	42
	Median	194	128	138
	(IQR)	(111–231)	(96–197)	(103–198)
NRS-H (≥3)	N	26	26	20
	Median	146	136	132
	(IQR)	(108–201)	(87–198)	(116–185)
SARC_F-L (<4)	N	82	82	59
	Median	180	130	135
	(IQR)	(111–226)	(96–198)	(106–194)
SARC_F-H (≥4)	N	4	4	3
	Median	164	122	121
	(IQR)	(109–215)	(69–315)	(91–273)

**Table 3:** Summary statistics of SII values across different measurement time points and stratified by NRS and SARC-F scores.

SII		1.	2.	3.
NRS-L (<3)	N	60	60	42
	Median	827	341	682
	(IQR)	(477–1170)	(176–701)	(443–1091)
NRS-H (≥3)	N	26	26	20
	Median	704	377	637
	(IQR)	(501–1187)	(284–704)	(469–1304)
SARC_F-L (<4)	N	82	82	59
	Median	824	343	659
	(IQR)	(471–1186)	(211–704)	(450–1104)
SARC_F-H (≥4)	N	4	4	3
	Median	812	467	593
	(IQR)	(596–1565)	(207–1799)	(485–2699)

## Data Availability

The data supporting the findings of this study are available from the corresponding author upon reasonable request.

## Abbreviations

The following abbreviations are used in this manuscript:

NLR	Neutrophil-to-Lymphocyte Ratio
PLR	Platelet-to-Lymphocyte
SII	Systemic immune-Inflammation Index
mCRC	Metastatic Colorectal Cancer (mCRC).
NRS	Nutrition Risk Screening
SARC-F	Strength, Assistance in walking, Rise from a chair, Climb stairs, Falls
ECOG	Eastern Cooperative Oncology Group Performance Status
MSCT	Multi-slice Computed Tomography
CBC	Complete Blood Count
NRS-L	Nutrition Risk Screening—Low Risk
NRS-H	Nutrition Risk Screening—High Risk
SARC_F-L	SARC-F—Low Risk
SARC_F-H	SARC-F—High Risk
RECIST	Response Evaluation Criteria in Solid Tumors
